# Genome-Wide Association between Transcription Factor Expression and Chromatin Accessibility Reveals Regulators of Chromatin Accessibility

**DOI:** 10.1371/journal.pcbi.1005311

**Published:** 2017-01-24

**Authors:** David Lamparter, Daniel Marbach, Rico Rueedi, Sven Bergmann, Zoltán Kutalik

**Affiliations:** 1 Department of Computational Biology, University of Lausanne, Lausanne, Switzerland; 2 Swiss institute of Bioinformatics, Lausanne, Switzerland; 3 Department of Integrative Biomedical Sciences, University of Cape Town, Cape Town, South Africa; 4 Institute of Social and Preventive Medicine (IUMSP), Lausanne University Hospital, Lausanne, Switzerland; Washington University in Saint Louis, UNITED STATES

## Abstract

To better understand genome regulation, it is important to uncover the role of transcription factors in the process of chromatin structure establishment and maintenance. Here we present a data-driven approach to systematically characterise transcription factors that are relevant for this process. Our method uses a linear mixed modelling approach to combine datasets of transcription factor binding motif enrichments in open chromatin and gene expression across the same set of cell lines. Applying this approach to the ENCODE dataset, we confirm already known and imply numerous novel transcription factors that play a role in the establishment or maintenance of open chromatin. In particular, our approach rediscovers many factors that have been annotated as pioneer factors.

## Author Summary

Transcription factor binding occurs mainly in regions of open chromatin. For many transcription factors, it is unclear whether binding is the cause or the consequence of open chromatin. Here, we used datasets on open chromatin and gene expression provided by the ENCODE project to predict which transcription factors drive transitions between open and closed states. A signature of such a factor is that its expression values are correlated to chromatin accessibility at its motif across the same set of cell lines. Our method assesses this correlation while accounting for the fact that some tested cell lines are more related than others. We find many transcription factors showing evidence of driving transitions and a high proportion of these transcription factors are known pioneer factors, i.e., they play a role in opening up closed chromatin.

## Introduction

In higher eukaryotes, certain sequence-specific transcription factors (TFs), which we will call *chromatin accessibility regulators* (CARs), are responsible for establishing and maintaining open chromatin configurations [1,2]. CARs therefore play a fundamental role in transcriptional regulation, because open chromatin configurations are necessary for additional TFs to bind and transcriptionally activate target genes.

CARs that can bind closed chromatin and open up chromatin are called pioneer TFs [3]. The comprehensive identification of pioneer TFs with high confidence still needs further research. While some pioneer TFs are well studied, others have only preliminary evidence, or are only computationally predicted. Some well studied examples include *FOXA1*, whose winged helix domains disrupt DNA–histone contacts, and *POU5F1*, *SOX2* and *KLF4*, which are used in production of induced pluripotent stem cells (iPSC) [4,5]. Further pioneer TFs such as *ASCL1*, *SPI1* and the *GATA* factors are used in transdifferentiation, and *PAX7* plays a role in pituitary melanotrope development [5–7]. However, not all pioneer TFs are involved in development and cell type conversions: the *CLOCK*-*BMAL1* heterodimer is part of the circadian clock and the tumour suppressor *TP53* is involved in the cell cycle, while its close homolog *TP63* is involved in skin development [8–10].

Recent studies suggest that maintaining open chromatin is a dynamic process with pioneer and other TFs binding and unbinding rapidly and continually recruiting additional chromatin remodelling factors that are not sequence specific [2,11,12]. TFs vary in their ability to recruit particular remodelling factors, for example the TFs *STAT5A/B* and *MYOG* motifs enrich in binding sites of the *SWI/SNF* remodelling complex but not in *ISWI* remodelling complex binding sites, whereas *YY1* motifs were found exclusively in *ISWI* complex binding sites [2]. A natural question then is which TFs are relevant to maintain open chromatin and can therefore be called CARs.

One approach to test whether a given TF is a CAR is to perform a knock-down of this TF followed by an open chromatin assay to see whether chromatin regions containing the respective motif preferentially change from open to closed [13]. However, this approach is very time consuming because it requires a separate knock-down experiment for each TF. To define pioneer TFs specifically, one can check if the TF has the ability to bind nucleosomal DNA *in vitro* and validate the results *in vivo* [14]. Recently, a computational method called *Protein interaction Quantification (PIQ)* has been published that aims to recover pioneer TFs by estimating both TF binding and ensuing chromatin changes from the same Dnase1 hypersensitivity (DHS) experiments [15]. However, *PIQ* did not predict some well known pioneer TFs such as *FOXA1*, *SOX2* and *POU5F1* showing that further improvements are possible [3].

Here we introduce a data driven approach to predict CARs. Our approach relies on the joint analysis of a large collection of DHS and coordinated gene expression data to estimate TF activity independently of DHS data. We first define the *motif accessibility score* for a given TF for each cell line based on the enrichment of its binding motif in regions with open chromatin. We then associate these scores with gene expression values across all available cell lines. This should allow us to predict which factors have a role either in establishment or maintenance of open chromatin, although it will not reveal which mode predominates (to determine this, further experiments will be necessary).

We used our approach on data generated as part of the ENCODE project [16,17]. This uncovered numerous TFs whose motif accessibility is robustly associated with mRNA expression across 109 cell lines suggesting either a role in the establishment or maintenance of open chromatin. Also, we see that our uncovered TFs are strongly enriched for known pioneer TFs. This suggests that the TFs we identified are good candidates for CARs.

## Results

### A linear mixed model approach to predict chromatin accessibility regulators

**Fig 1:**
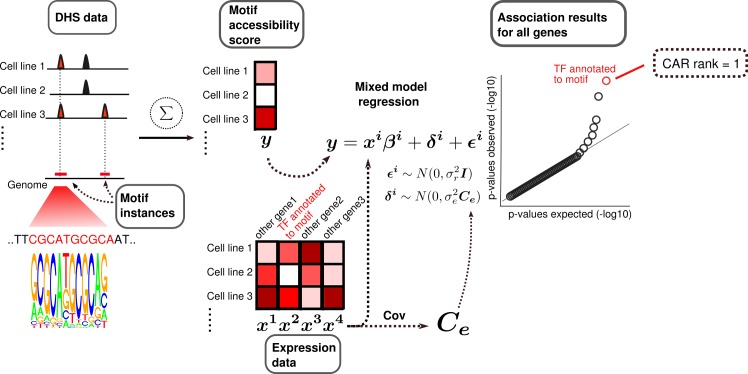
Mixed model approach for identification of chromatin accessibility regulators. For a TF binding motif, we search for all its instances in the genome. For each cell line, we calculate the accessibility score by counting how many motif instances are found in the open chromatin fraction of the genome. After further normalization, these accessibility scores are compared to gene expression values for all genes via regression (Methods). To account for confounding, we use mixed model regression, where an additional random component is used with the same covariance structure as the gene expression matrix. To be considered a CAR candidate, motif accessibility of a TF must show strong association (low p-value) with the expression of the corresponding TF gene compared to other genes. The gene-level *CAR rank* of a TF is defined as the rank of its association p-value among the p-values for all genes.

Our approach rests on the assumption that the activity of a CAR is correlated with the amount of open chromatin in the vicinity of its potential binding sites. Both quantities can be estimated from genomic data: For the CAR activity we use its gene expression level as a proxy for the active protein concentration. The effect of this activity is approximated by the open chromatin fraction of the genome around its binding motif instances (Fig 1). Specifically, we count the number of instances of the binding motif of a given TF in the open chromatin fraction of the genome to define a motif accessibility score. A naive approach would be to use standard linear regression between the motif accessibility score and the expression level of a given TF to identify CAR candidates. Yet, this method has an elevated type I error rate, as it does not account for confounding due to cell line relatedness or batch effects. To overcome this limitation, we use here a linear mixed model (LMM) framework, where a random effect accounts for such confounding factors (which has been shown to work well in genetic association studies [18–20]). For a given motif, we use the linear mixed model framework to find the association p-value between its accessibility score and the measured expression of the TF gene. We then compare this p-value to the p-values calculated using the measured expression of each of the other genes as regressors. If confounding is controlled for, most association p-values should follow a uniform [0,1] distribution. Furthermore, if the TF is a CAR, its p-value should be low compared to other genes. We thus define the *CAR rank* of a TF as the rank of its association p-value among all genes (see example in Fig 1). Low CAR ranks indicate strong association between motif accessibility and TF expression, suggesting that the TF is a CAR.

**Fig 2:**
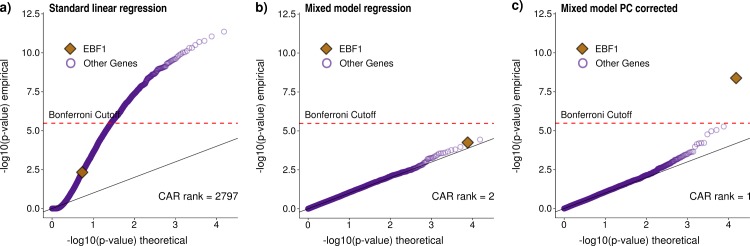
Association between motif accessibility and mRNA expression for the putative chromatin accessibility regulator *EBF1*. Three different regression models (a-c) were used to compute association p-values between the accessibility of a given TF motif (here *EBF1*) and mRNA expression for each of the assayed 15K protein-coding genes. Results are visualized as qq-plots showing the -log10 transformed p-values. (a) Association p-values obtained using standard linear regression. Due to confounding, p-values are strongly inflated and *EBF1* motif accessibility does not show strong association with *EBF1* expression compared to other genes. (b) The linear mixed model (LMM) successfully corrects for confounding, with most p-values following the null distribution as expected. The association between *EBF1* motif accessibility and *EBF1* expression now ranks second among all genes and first among all TFs, although it does not pass the Bonferroni significance threshold. (c) Additionally controlling for the first principal component of the motif accessibility matrix corrects for a strong batch effect (Methods), which further improves the signal. Using this approach, *EBF1* motif accessibility showed the strongest association precisely with *EBF1* expression (i.e., the gene-level CAR rank equals one), suggesting that *EBF1* may be a CAR, in agreement with the literature [22]. As a further illustration for the improvements achieved using the mixed model approach S1 Fig shows the analogous plot for FOXA1, the first discovered pioneer factor [4,5].

Specifically, we used DHS data as well as mRNA expression data across 109 cell lines. To calculate motif accessibility scores we used 325 TF binding motifs from the HOCOMOCO database [21]. As expected, we observed severe confounding when using standard linear regression, which was controlled using linear mixed effect model regression (Fig 2).

### ChIP-seq shows widespread binding of homologous TFs to each other's motifs

**Fig 3:**
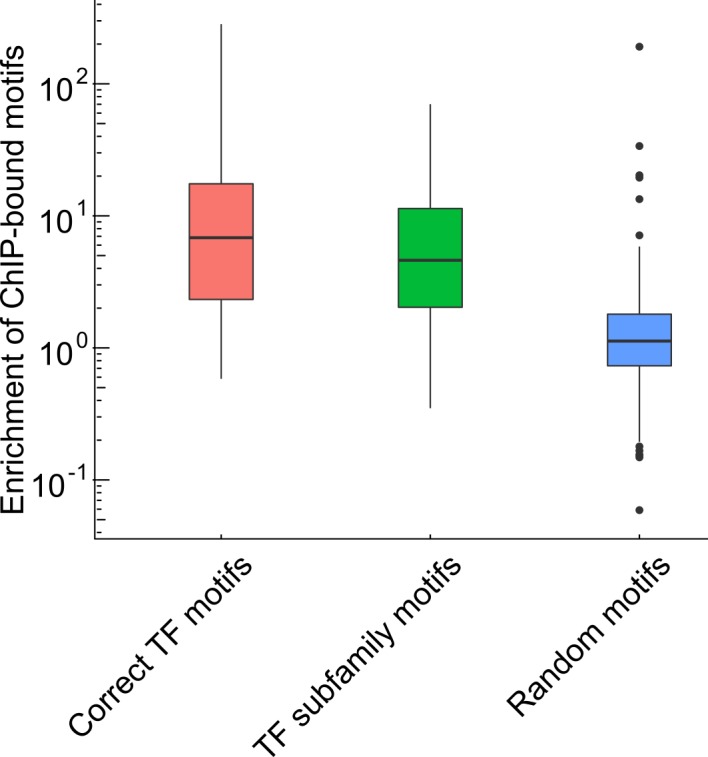
Enrichment of bound motifs for a given TF and its subfamily members. All TF ChIP-seq experiments from the Myers-lab released as part of the ENCODE project were downloaded. For each TF ChIP-seq experiment we also obtained the corresponding TF motif from the HOCOMOCO database [25]. For a given ChIP-seq experiment, we looked at the processed DHS peaks in the same cell line. We partitioned DHS peaks into two groups depending on whether they were bound by the TF (overlap with a ChIP-seq peak) or not. We then calculated both the fraction of bound and unbound DHS peaks containing a given motif. The enrichment of bound motifs was defined as the ratio of these two fractions. Results are shown from left to right for: the motifs of the TFs that were assayed in the corresponding ChIP-seq experiments (Correct TF motifs), motifs of other TFs from the same subfamily (TF subfamily motifs), and randomly sampled motifs (Random motifs). During sampling, each motif was sampled as often as the number of ChiP-seq experiment available for that motif. We see strong enrichment of TF motifs in ChIP-seq peaks of the TF as well as its subfamily members.

Our method relies on TF motif accessibility and expression data to predict CARs. However, evolutionarily related TFs have similar binding motifs [23]. Motif accessibility may therefore associate not only with the expression of the annotated TF, but also with the expression of a homologous TF with a similar motif. Therefore, we mapped TFs into subfamilies using the homology-based clustering TFClass [24]. The 1,557 TFs were grouped into 397 subfamilies. Using a collection of 329 ChIP-seq profiles from ENCODE, we saw strong enrichment of TF motifs in ChIP-seq peaks of the TF as well as its subfamily members (Fig 3). We therefore consider any strong association between a motif and a member of the subfamily of its TF as a signal for a CAR.

### Comprehensive prediction of chromatin accessibility regulators

Next, we used the linear mixed model strategy to predict CARs among TFs. We used 325 motifs from HOCOMOCO (after filtering motifs showing low overlap with DHS signal, see Methods). For each motif, we used a linear mixed effect model to compute its association with mRNA expression for 1,188 known TFs. Due to the redundancy of motifs within the same TF subfamily (see preceding section), we also computed CAR ranks at the level of TF subfamilies. To this end, we retained the most significant association p-value within each subfamily corrected for subfamily size (see Methods and S2 Fig). Under the null model (when TFs are not CARs), CAR ranks should be uniformly distributed across all subfamilies, so that deviation from uniformity indicates presence of CARs.

**Fig 4:**
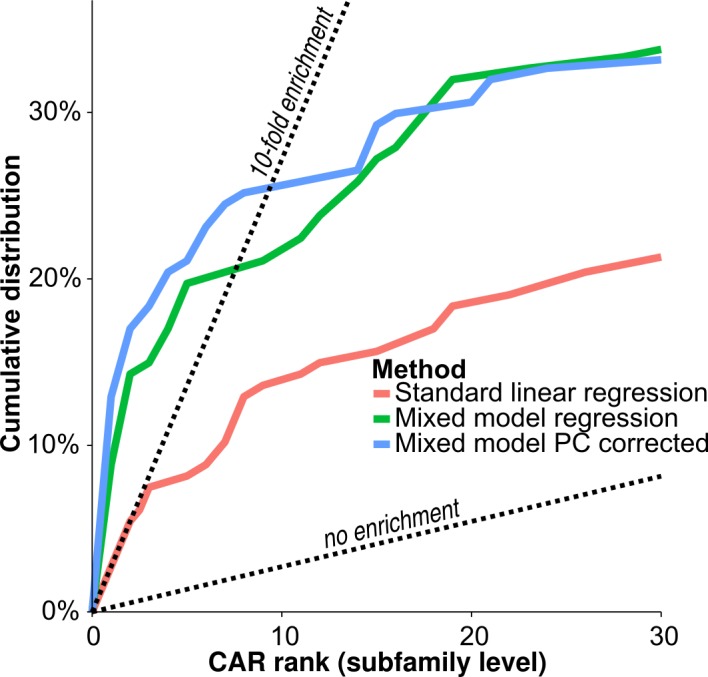
Method comparison across all subfamilies. Cumulative distribution of *CAR* ranks at the subfamily level for the 147 tested subfamilies using the three different modelling strategies: ‘standard linear regression’, ‘mixed model regression’ and ‘mixed model PC corrected’ (see legend of Fig 2 and Methods). We see strong enrichment of low ranks implying deviation from the null hypothesis. The linear mixed modelling increases enrichment of low CAR ranks.

We found strong enrichment of low CAR ranks at the subfamily level (Fig 4, S1 Table). The enrichment was stronger when using mixed modelling instead of standard linear regression, underlining again the importance of proper control for confounding factors. When looking at the threshold that leads to 10-fold enrichment of low CAR ranks compared to uniformity (i.e., 10% false discovery rate), we found that 25% of all subfamilies have a CAR rank that falls below that threshold. These results show that many TFs do have an impact on the open chromatin fraction and can be defined as CARs.

To validate our results based on the ENCODE dataset, we applied our CAR calling strategy to data from another large scale effort, the ROADMAP Epigenomics consortium [26]. Coordinated open chromatin and expression data have been released for 56 samples. For 29 of these samples, open chromatin was assayed directly. For the other samples, open chromatin information was imputed from other available epigenetic measurements. The ROADMAP collection is derived mainly from human tissue samples and primary cell lines (whereas ENCODE is biased towards immortalized cell lines). Further differences are that expression was measured using RNA sequencing. We applied our method to these datasets and compared results to the results derived in ENCODE. Most subfamilies predicted to be CARs in ROADMAP were recovered in ENCODE (see S3 Fig). Furthermore, while subfamilies predicted to be CARs in ENCODE showed enrichment for low CAR ranks in ROADMAP, subfamilies not predicted to be CARs in ENCODE did not show enrichment for low CAR ranks in ROADMAP (see S4 Fig). These results are concordant with both datasets, pointing toward the same factors being CARs and the higher power of the ENCODE data to detect CARs, potentially due to higher sample size, reliance on direct measurements of DHS and lower fraction of complex tissue samples.

To evaluate the impact of the motif search strategy, we investigated the robustness of the pipeline with respect to the motif search. Results were stable and power was only affected by varying motif cutoffs (S5 Fig, S6 Fig). Additionally, we investigated whether choosing the cutoff based on ChIP-seq data changed results. For each TF with available ChIP-seq data, we used an individual cutoff such that all called binding sites have fixed true positive rate (using the ChIP-seq data as the ground truth). Again, results were stable no matter how the cutoff was assigned (S7 Fig and S8 Fig).

### Pioneer TF subfamilies enrich in predicted chromatin accessibility regulators

**Fig 5:**
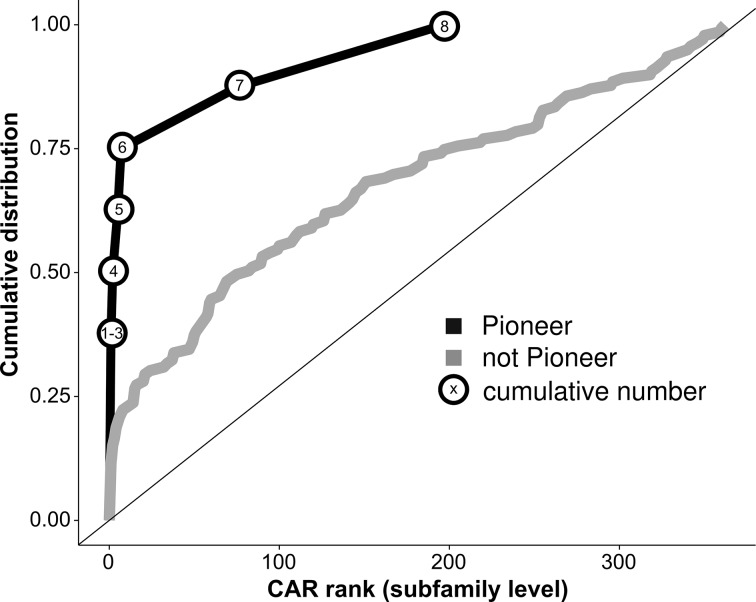
Known pioneer TF subfamilies strongly enrich in predicted chromatin accessibility regulators. Shown in grey is a scaled cumulative distribution plot for subfamily level CAR ranks of subfamilies not annotated as pioneers in Iwafuchi-Doi et al. [3]. In black, we see the cumulative number of pioneer subfamilies that reached at least a given CAR rank. Six out of eight subfamilies show a low CAR rank, which is more than three times as many as one would expect on average when sampling from non-pioneer subfamilies.

As mentioned above, one well-defined class of CARs are pioneer TFs that can bind and open closed chromatin. Therefore, subfamilies annotated to known pioneer TFs should have low CAR ranks. To test enrichment formally, we used a recently published list of established pioneer TF subfamilies (Methods) [3]. We asked whether these subfamilies were predicted as CARs using our methodology. For eight subfamilies in the list for which we had the motif, six showed at least ten-fold enrichment (i.e. having a CAR rank at the subfamily level below ten) (Fig 5). To assess significance, we used the Wilcoxon ranksum test leading to a p-value of 0.0087. When using the hypergeometric test with 10-fold enrichment cutoff (Fig 4), the p-value was even lower (P = 0.0016). Because our approach to uncover CARs is biased towards TFs with large mRNA expression variability (S9 Fig), we sought to control for potential confounding introduced by the fact that the tested pioneer factors might also have large expression variability. Controlling for expression variability only slightly increased the p-values from 0.0087 to 0.024 and from 0.0016 to 0.0027, respectively.

### Downstream genes can show strong associations for activating chromatin accessibility regulators

It is known that the activity of some TFs is mainly regulated by the level of their cofactors rather than their own protein concentration [27]. These TFs are often present in their inactive form in the cell, which can then be quickly activated upon binding of the cofactor. This allows the cell to rapidly respond to environmental cues. An example of this phenomenon are steroid receptor TFs, which initiate transcriptional changes upon steroid hormone binding [28]. In such cases, one would not expect a strong association between the mRNA expression level of a receptor TF and its motif accessibility because mRNA expression would rather be correlated to the amounts of inactive TF protein in the cell, while TF activity should depend on the strength of the environmental stimulus. However, if the TF strongly activates mRNA expression of other genes, it might be possible to predict whether the TF is a chromatin accessibility regulator by looking at associations between the motif accessibility of the TF and the expression of its downstream genes.

**Fig 6:**
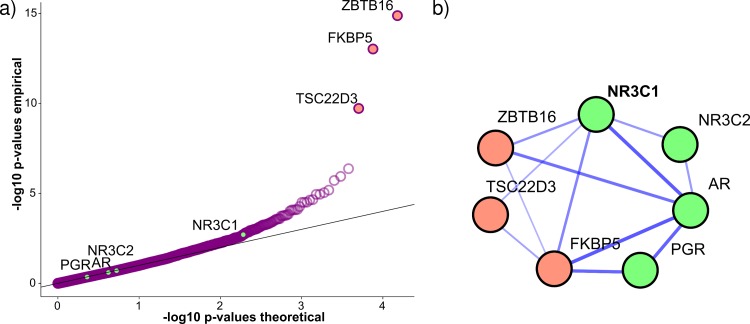
Strong associations between GR-like receptor motif and glucocorticoid response genes. a) Association results for motif accessibility of the TF *NR3C1*, which belongs to the GR-like receptor subfamily, and mRNA expression across all genes. -Log10 transformed p-values are shown in a QQ-plot. *NR3C1* motif accessibility shows strong association with mRNA expression of three glucocorticoid response genes (orange), but only weak association with expression of *NR3C1* and other GR-like receptor TFs (green). In this example, motif accessibility is strongly associated with downstream gene expression, but only weakly with expression of the TF itself. b) The network shows functional relationships among the GR-like receptor TFs (green) and the three most strongly associated genes (orange), which are all glucocorticoid response genes. The strength of links shows confidence in functional relationship given in the *STRING* database. We see numerous links between the downstream glucocorticoid response genes and the GR-like receptor TFs in the STRING database, confirming their functional relatedness, where *NR3C1* has the most links to associated genes.

To explore this strategy, we looked at associations across all genes and motifs that were below the overall Bonferroni threshold (9.6 x 10^−9^). For five out of 13 such motifs, members of the corresponding subfamily had top scores. In four further cases, a gene from a TF subfamily was ranked close to the top that was highly related (i.e. part of the same family [24]) to the motifs’ corresponding subfamily but not identical with it. This suggests that the TF subfamily clustering was too fine-grained in these cases. Surprisingly, for one motif, the significant association had a negative effect size (the negative association was observed between NUDT11 and the motif for RARG), which might reflect an indirect effect. The remaining three motifs were all annotated to the GR-like receptors, which encompass four TFs (AR, NR3C1, NR3C2, PGR). The accessibilities of these three motifs all associated strongly with the expression of three genes (FKBP5, ZBTB1, TSC22D3). When using the STRING database to check for functional links between these genes, all genes had links to a GR-like receptor (Fig 6) [29]. In fact, all three genes are known to be glucocorticoid response genes. These results suggest that some GR-like receptors might act as a CAR. For strongly activating factors, the power of the analysis can therefore be strengthened by incorporating results from downstream genes.

## Discussion

It is well known that TF binding correlates with open chromatin [17]. However, for many TFs, it is not clear whether their binding is the cause or the consequence of open chromatin. Here, we used datasets provided by ENCODE to predict chromatin accessibility regulator candidates, i.e., TFs that are able to establish or maintain open chromatin configurations. We devised an approach using linear mixed models to deal with the extensive confounding that one encounters in genome-wide data from heterogeneous sources. Our method uncovers a set of TFs whose expression is associated with their motif accessibility, suggesting a role in maintenance of an open chromatin configuration.

Potentially our methodology could be extended to histone modification data instead of DHS data. We applied our method to H3K4me3 data for cell-lines but did not see strong enrichment (S10 Fig).

Because pioneer TFs are by definition CARs, our predictions should be enriched for known pioneer TFs. We tested this formally for a list of pioneer TF subfamilies recently published by Iwafuchi-Doi et al. [3]. Six out of eight pioneer subfamilies were indeed predicted by our method to be CARs: *FOXA1*, *GATA6*, *KLF4*, SOX2, *SPI1* and *TP63* were the pioneer TFs driving these signals. The two subfamilies not predicted to be CARs were *POU5* and *CLOCK*. *SOX2* was the gene most strongly associated with POU5F1 motif accessibility with a low p-value of 5 x 10^−6^ (S11 Fig). *POU5F*1 acts together with *SOX2* to maintain undifferentiated states [30]. The two TFs also physically interact and a recent study proposed a model where *SOX2* guides *POU5F1* to target sites [31]. The *CLOCK* subfamily members have a role in the cell cycle, acting as TFs for the circadian pacemakers [32]. It is possible that average mRNA expression of these TFs in unsynchronized cell lines is not a meaningful measure for their activity. In addition to the eight aforementioned factors we found further factors discussed in the pioneer TF literature such as *TFAP2C*, *EBF1*, *CEBPD/B*, *OTX2*, *NFKB* and *STAT5* (Table 1) [22,33–37]. In addition, when combining our predictions with those from the PIQ method[15], we observed substantial performance improvement compared to either method alone (S12 Fig).

**Table 1:** Predicted pioneer factors. Shown are the CAR ranks of factor subfamilies that were discussed in the main text. These included subfamilies labelled pioneers in [3] and consequently used as a member of the true positive set used in Fig 5 (these subfamilies are set in bold face). Additionally, subfamilies are shown that are predicted to be CARs and for which there exist limited literature evidence for pioneer activity. For each subfamily, the top-scoring gene among all genes in the subfamily is mentioned. A complete table for all tested subfamilies is given in S1 Table.

Subfamily name	Top gene in subfamily	CAR rank (subfamily level)	Pioneer evidence
C/EBP	*CEBPD*	1	[34]
**AP-2**	*TFAP2C*	1	[3,33]
**Krüppel-like factors**	*KLF4*	1	[3,4]
**FOXA**	*FOXA1*	1	[3–5]
**Group B**	*SOX2*	1	[3,4]
NF-kappaB p65 subunit-like factors	*RELB*	1	[38]
Early B-Cell Factor-related factors	*EBF1*	1	[22]
**Two zinc-finger GATA factors**	*GATA6*	2	[3,5]
STAT factors	*STAT5B*	2	[37]
OTX	*OTX2*	3	[35]
**Spi-like factors**	*SPI1*	5	[1,7]
**Arnt-like factors**	*ARNTL2*	76	[3]
**POU5 (Oct-3/4-like factors)**	*POU5F1*	197	[3,4]

One limitation of our approach is that it cannot discern between open chromatin establishing TFs and open chromatin maintaining TFs. A way to discern the relative roles could be to perform overexpression and knock-down experiments followed by an open chromatin assay for the TFs found by our approach. While this is out of the scope for the current study, we hope that our method can help in prioritizing such experimental efforts.

Further, by its very nature, our methodology cannot with certainty resolve between TFs that belong to the same sub-family. It shares this weakness with almost any method relying on TF motifs. The procedure associates the expression values of each TF separately to the motif accessibilities and one strong association is enough to lead to low CAR ranks for the subfamily. The TF in the subfamily whose expression is the most strongly associated to one of the subfamily motif is naturally also the strongest candidate for CAR activity. (This information is given in Table 1 as well as in S1 Table). However, if the expression values of the subfamily members are also strongly correlated, we cannot be sure which ones are driving the association.

It is also clear that multiple conditions have to be met for the approach to work. First and foremost, mRNA expression has to be correlated sufficiently with protein concentration of the CAR. Typically, only a fraction of the variation in protein concentration can be explained by variation in mRNA abundances [39]. Nevertheless, better power of our approach can always be achieved by increasing sample size, as long as there is at least some correlation. Further, it is reasonable to assume that our approach will perform better on TFs with a large dynamic range across cell types. This seems indeed to be the case, since most TFs predicted to be CARs tend to have large mRNA expression variance (S9 Fig). Sampling more and diverse cell lines could address this issue, because it should increase the dynamic range.

This restriction would also suggest that our approach is biased against cell type specific TFs. However, when looking at tissue expression patterns (www.gtexportal.org [40]) of the predicted CARs, we found both: TFs that showed expression in a large proportion of cell lines such as *EBF1* and *STAT5B* as well as quite specific TFs. Examples of specific CARs are *SPI1*, which only showed expression in whole blood, and *OTX2*, which only showed expression in some brain regions. It is possible that the use of immortalized cell lines leads to larger gene expression variability in the sample facilitating the detection of such tissue-specific CARs.

For some TFs, activity mainly depends on cofactors. For example, for steroid hormone receptors, hormone molecules activate a pool of inactive TF already present in the cell. In such cases measuring TF activity with gene expression measures can be misleading and one would not expect an association between the expression of a TF and the accessibility of its motif. For example, for the accessibility score of *NR3C1*, we saw much stronger associations with the expression levels of a small set of glucocorticoid response genes (*ZBTB16*, *FKBP5*, *TSC22D3)* than that of *NRC1* itself [41–43]. This difference in signal strength is in line with the activity of *NR3C1* being mainly regulated by glucocorticoid binding and not *NR3C1* gene expression levels. Of note, *NR3C1* was reported to have pioneer activity [1].

In summary, we exploited the rich data source of ENCODE to find TFs whose mRNA expression levels are directly linked to the open chromatin fraction of the genome. Although our approach in its current form is able to find TFs with strong associations, it is also clear that increasing power by adding more cell lines would find more TFs with an association. From the current data, we would estimate that at least 25% of TF subfamilies show a low CAR rank at the subfamily level, suggesting that the regulation of chromatin accessibility is a pervasive phenomenon amongst TFs.

## Materials and Methods

### Motif accessibility score creation

Annotated open chromatin (FDR <0.01) peaks were downloaded from the EBI website (see URL section) and trimmed to the top 90,000 peaks for each cell line. 426 motifs were downloaded from the HOCOMOCO website and aligned to the reference genome with FIMO [21,44]. Motif occurrences with a p-value below 10^−5^ were kept for processing. For each motif, we counted the number of DHS peaks overlapping a motif instance in a given cell line using bedops [45]. Results were filtered to motifs that were present in at least 150 DHS peaks on average, leaving 344 motifs. For a given motif, we quantile-normalized the values to follow a normal distribution yielding the raw motif-activity matrix with rows corresponding to motifs and columns corresponding to cell lines. The resulting matrix was iteratively scaled to zero mean and unit standard deviation, first row-wise (across cell lines) then column-wise, until convergence [46,47]. Next, we saw that the cell-line wise covariance matrix had a very large first eigenvalue, with a corresponding eigenvector that did not track well the different tissue origins of the various cell lines. Assuming that this leading principal component largely captured batch effects, we chose to regress out the first eigenvector from each row of the matrix, leading to better agreement between expression and motif accessibility correlation matrices (S13 Fig). After this step, we quantile-normalized the data per motif to follow a normal distribution to ensure that the assumptions of the applied statistical model were met. To map motifs to TFs and TF subfamilies, we used the *TfClass* hierarchy [24]. Of the 344 tested motifs, we mapped 330 to a TF and its subfamily. Of these, 325 had expression data available for a subfamily member.

### Expression matrix creation

We downloaded raw expression microarray data from the GEO repository (GSE1909 and GSE15805). (ENCODE micro-array data was used instead of RNA-seq because to-date more cell lines with DHS information have also RNA expression measured by micro-array than RNA-seq). We background corrected and normalized using the RMA-algorithm implemented in the oligo package to process all arrays for which DHS data was also available [48,49]. Only the core set data was used. The data were summarized to gene level [50]. Only results that had a one-to-one mapping between genes and gene probesets were kept. 15,119 genes could be annotated in this fashion. Because for many cell lines more than one experiment was conducted, we summarized multiple plates by averaging gene results across experiments. The resulting matrix was iteratively scaled to zero mean and unit standard deviation, first row-wise (across cell lines) then column-wise, until convergence [46,47].

### Linear mixed effect model

The model proposed is

$$y=x_{i}\beta^{i}+\delta^{i}+\varepsilon^{i}.$$

Where *y* is a vector of motif accessibility scores across *n* cell lines, *x*_*i*_ is the expression vector of gene, *i*, *β*^*i*^ is the effect size of gene *i*:

$$\varepsilon^{i}{∼N}_{n}(0,\sigma_{r}^{2}I_{n})$$

and

$$\delta^{i}∼N_{n}(0,\sigma_{e}^{2}C_{e}).$$

*C*_*e*_ is the covariance matrix of the *n x p* expression matrix:

$$C_{e}=\frac{1}{p}\sum_{i=1}^{p}x_{i}x_{i}^{T}.$$

For each gene *i*, *β*^*i*^, *σ*_*r*_, and *σ*_*e*_ are estimated via maximum likelihood and the null hypothesis *β*^*i*^ *=* 0 is tested via a likelihood ratio test [18,20]. More details on this procedure are given in S1 Appendix.

### Calculating CAR ranks at the subfamily level

For each motif in HOCOMOCO, we used the mixed model association results across all 1,188 known TFs for which we had mRNA expression data [21]. This yielded a matrix of association p-values for all pairs of 325 motifs (belonging to 147 *TFClass* subfamilies) and 1188 TFs (belonging to 368 *TFClass* subfamilies). Due to the fact that homologous TFs have similar binding motifs, we sought to aggregate results into CAR *ranks* at the subfamily level (S1 Fig). To achieve this, we reduced the 325 x 1188 motif-TF association matrix to a 147 x 368 matrix of associations between motif subfamilies and TF subfamilies. In practice, for each motif subfamily-TF subfamily pair we collected the most significant p-value among all motif-TF pairs in these subfamilies and multiplied it with the total number of such motif-TF pairs to correct for subfamily size. Finally, for each motif *subfamily*, we ranked the adjusted p-values across all TF subfamilies and defined its *CAR rank* as the rank of its corresponding TF subfamily.

### Calculating pioneer subfamily enrichment

To get an external annotation of pioneer factors, we used a recently published list of established and predicted pioneer factors (Table 1 in Iwafuchi-Doi et al. [3]). We used a hypergeometric test at the 10-fold enrichment cut-off (Fig 4), as well as a ranksum enrichment test. To derive a ranksum statistic, we summed the CAR ranks of the eight subfamilies annotated as pioneers. To assess significance of this statistic, we used permutation tests: For each of the 50,000 permutation samples, we picked eight CAR ranks from the set of subfamilies not annotated as pioneers and summed them to derive 50,000 permutation sample statistics. The p-value was approximated as the fraction of permutation sample statistics of greater or equal size as the statistic derived for the annotated pioneers. To control pioneer enrichment for mRNA expression variation, we first calculated the expression variance of each TF across all cell lines. The distribution of variance values was transformed to follow a standard normal distribution. We then used the maximal expression variance observed for any TF in each subfamily. To assess significance, we used permutation tests: we sampled eight non-pioneer subfamily level CAR ranks 50,000 times. However, subfamilies were not sampled uniformly: We sampled four non-pioneer subfamilies with maximal expression variance between the 0th and the 50th quantile of the eight pioneer subfamilies, and four non-pioneer subfamilies with maximal expression variance between the 50th and the 100th quantile of the eight pioneer subfamilies.

### Processing ROADMAP data

RNA-seq data were downloaded from the ROADMAP website (see section ‘URLS’) for 56 cell lines. We used only genes with average read count above 50, which removed 12% of genes. The number of reads plus a pseudo-count of one to were log-transformed. Samples were then quantile normalized to the average mean distribution [51]. The resulting matrix was iteratively scaled to zero mean and unit standard deviation, first row-wise (across cell lines) then column-wise, until convergence [46,47].

To derive motif accessibility scores, imputed DHS data were downloaded for 56 cell lines from the ROADMAP website (see section ‘URLs’). From these datasets motif accessibility scores were derived in the same fashion as for the ENCODE DHS data. To derive CAR ranks, the same strategy was employed as for the ENCODE dataset.

### Defining ChiP-seq guided motif cutoff

To compare fixed motif cutoffs to a variable motif cutoff guided by ChIP-seq, the following procedure was used. ChIP-seq data from the Myers and Snyder lab in the ENCODE collection for which dnase1 and expression data were available were downloaded and each ChiP-seq experiment was mapped to a dnase1 experiment based on cell line and to the motif of the TF, yielding mappings to 75 motifs (belonging to 50 subfamilies). For a given motif and cell line pair for which ChIP-seq data (as well as DHS data) was available, each DHS region was annotated with the p-value of its most significant motif instance (given that they contained a motif with p-value below 5x10^-5^) as well as whether it overlapped with a ChIP-seq peak. The motif p-value cutoff was defined such that a fixed fraction of peaks with motifs below that cutoff would validate in the ChIP-seq experiment. Three true positive rates were chosen for this comparison 0.3, 0.5 and 0.7 (see S7 Fig, S8 Fig). Only experiments were used for which it was possible to choose a motif cutoff such that the highest validation rate (i.e. 0.7) could be reached. If multiple ChIP-seq experiments were available per motif, the median p-value cutoff was chosen for each validation rate. We compared these strategies using a fixed cutoff for all motifs of 10^−5^, which was used throughout the rest of the paper. Results obtained are similar when using ChIP-seq guided cutoffs or fixed cutoffs.

### URLs

Code for reproduction (including scripts for data download) is available at: **https://github.com/dlampart/csrproject**

ENCODE DHS peaks were downloaded from: **http://ftp.ebi.ac.uk/pub/databases/ensembl/encode/integration_data_jan2011/byDataType/openchrom/jan2011/fdrPeaks/**

ROADMAP expression data were downloaded from: **http://egg2.wustl.edu/roadmap/data/byDataType/rna/expression/57epigenomes.N.pc.gz**

ROADMAP imputed DHS peaks were downloaded from: **http://egg2.wustl.edu/roadmap/data/byFileType/peaks/consolidatedImputed/narrowPeak/**

ENCODE histone files were downloaded from: **ftp://hgdownload.cse.ucsc.edu/goldenPath/hg19/encodeDCC/wgEncodeUwHistone/**

## Supporting Information

S1 Appendix Supporting methods. (PDF)

S1 Table Results for comprehensive prediction of chromatin accessibility regulators. The table shows CAR ranks at subfamily level for each motif in the HOCOMOCO library. Subfamily identifiers correspond to the identifier used in *TFClass*. Additionally, the gene in the annotated subfamily with the highest gene wise CAR rank is given.
(XLSX)

S1 Fig Association between motif accessibility and mRNA expression for the bona fide pioneer factor *FOXA1*. Three different regression models (a-c) were used to compute association p-values between the accessibility of a given TF motif (here *FOXA1*) and mRNA expression for each of the assayed 15K protein-coding genes. Results are visualized as QQ-plots showing the–log10 transformed p-values. (a) Association p-values obtained using standard linear regression. Due to confounding, p-values are strongly inflated and *FOXA1* motif accessibility shows only mild association with *FOXA1* expression compared to other genes. (b) The linear mixed model (LMM) successfully corrects for confounding, with most p-values following the null distribution as expected. The association between *FOXA1* motif accessibility and *FOXA1* expression now ranks second among all genes and first among all TFs, although it does not pass the Bonferroni significance threshold. (c) Additionally controlling for the first principal component of the motif accessibility matrix corrects for a strong batch effect (Methods) and further lowers the CAR rank. Using this approach, *FOXA1* motif accessibility showed the strongest association precisely with *FOXA1* expression (i.e., the gene-level CAR rank equals one), in line with literature on *FOXA1* being a pioneer factor(Cirillo et al. 2002)(Cirillo et al. 2002; Soufi et al. 2015).
(PNG)

S2 Fig Overview of procedure to calculate *CAR* ranks on the subfamily level. We cluster TFs and motifs according to subfamily definitions given in *TFClass*. For each bicluster, we define the bicluster score as the most significant p-value between any TF and motif members of the bicluster corrected for bicluster size. We then rank bicluster scores across the TF subfamilies. If the bicluster joining a TF cluster and its corresponding motifs is ranked low, this is an indication of CAR activity.
(PNG)

S3 Fig CARs predicted from ENCODE data enrich in subfamilies with low CAR ranks in the ROADMAP dataset. DHS and expression data available for 56 samples (29 with assayed DHS and 27 with imputed DHS) as part of the ROADMAP data collection were used to predict CARs. Shown are CAR enrichment curves for ENCODE results stratified by CAR ranks derived from ROADMAP. Displayed are the following strata: ROADMAP CAR rank <10 (N = 9 observations in total), ROADMAP CAR rank <20 (N = 20 observations in total), ROADMAP CAR rank <30 (N = 25 observations in total), ROADMAP CAR rank <60 (N = 38 observations in total), ROADMAP CAR rank <100 (N = 58 observations in total), ROADMAP CAR rank > = 100 (N = 86 observations in total). We see that subfamilies with low ROADMAP CAR rank also tend to be predicted to be CARs when using the ENCODE data. This enrichment gets weaker for subfamilies with lower ROADMAP CAR ranking.
(PNG)

S4 Fig CARs ranks from ROADMAP data enrich only in subfamilies predicted to be CARs in ENCODE. DHS and expression data, available as part of the ROADMAP data collection, were used to predict CARs. Shown are CAR enrichment curves for ROADMAP results stratified by CAR predictions derived from ENCODE. Displayed are the following strata: ENCODE CAR rank <10 (N = 37 observations in total), ENCODE CAR rank > = 10 (N = 107 observations in total). While we see enrichment for low ROADMAP CAR rank in subfamilies predicted to be CARs via the ENCODE data, we see no enrichment in low ROADMAP CAR ranks for other subfamilies.
(PNG)

S5 Fig CAR detection power is stable to changes in motif cutoffs. Cumulative distribution of CAR ranks at the subfamily level using the three different motif cutoffs: 10^−5^ (used throughout the paper) is compared to 10^−6^ (yielding 9.3 fewer motifs on average [median]) and 5*10^−5^ (yielding 5.2 more motifs assigned on average). For each setting, we filtered motifs that did not overlap at least 150 DHS regions per cell line on average. Only subfamilies passing this filter in all settings were included (62 subfamilies in total). Power mildly increased at low CAR ranks for more stringent cutoffs at the cost of fewer motifs passing filtering. However, at false discovery rate of 10% power was nearly identical.
(PNG)

S6 Fig CAR prediction is stable with respect to changes in motif cutoffs. Shown are pairwise comparisons of different motif cutoffs. For each cutoff we derived CAR ranks for all tested subfamilies yielding one CAR rank list per cutoff. Pairwise comparisons of these lists were performed in the following manner: For each pair of rank lists, the first list was used to split the tested subfamilies into a ‘CAR set’ and its complement based on whether a subfamily had CAR rank below 10. For the second results list, two separate CAR enrichment curves were drawn, one curve for the ‘CAR set’ defined via the first list (black) and its complement (grey). Rows denote the cutoff used to derive the ‘CAR set’ and columns denote the cutoff used to draw the enrichment curves. For each setting, we filtered motifs that did not overlap at least 150 DHS regions per cell line on average. Only subfamilies passing this filter in all settings were included (62 subfamilies in total). We see that CARs predicted are stable with respect to varying motif cutoffs.
(PNG)

S7 Fig CAR detection power does not improve systematically when guiding motif cutoffs via ChIP-seq. Shown are cumulative distribution of CAR ranks at the subfamily level comparing fixed motif cutoff of 10^−5^ (used throughout the paper) is compared to variable motif cutoffs guided by ChIP-seq data, where motif cutoffs are adjusted such that called binding sites (i.e. DHS sites containing a motif instance) have a fixed validation rate compared to a gold standard defined by ChiP-seq. Chosen validation rates are 0.3, 0.5 and 0.7. For each setting, we filtered motifs that did not overlap at least 150 DHS regions per cell line on average. Only subfamilies passing this filter in all settings were included (32 subfamilies in total). While we see some variation in power, the variation is not systematic.
(PNG)

S8 Fig ChiP-seq data guiding motif cutoffs yields similar CAR predictions as regular motif cutoff. Shown are pairwise comparisons of different motif cutoff methods. For each cutoff method we derived CAR ranks for all tested subfamilies yielding one CAR rank list per method. Pairwise comparisons of these lists were performed in the following manner: For each pair of rank lists, the first list was used to split the tested subfamilies into a ‘CAR set’ and its complement based on whether a subfamily had CAR rank below 10. For the second results list, two separate CAR enrichment curves were drawn, one curve for the ‘CAR set’ defined via the first list (black) and its complement (grey). Rows denote the cutoff method used to derive the ‘CAR set’ and columns denote the cutoff method used to draw the enrichment curves. A fixed motif cutoff of 10^−5^ (also used throughout the paper) is compared to variable motif cutoffs guided by ChIP-seq data, where motif cutoffs are adjusted such that called binding sites (i.e. DHS sites containing a motif instance) have a fixed validation rate when compared to ChiP-seq. Chosen validation rates are 0.3, 0.5 and 0.7. For each setting, we filtered motifs that did not overlap at least 150 DHS regions per cell line on average. Only subfamilies passing this filter in all settings were included (32 subfamilies in total). We see that CARs predicted are stable with respect to varying motif cutoffs.
(PNG)

S9 Fig Predicted chromatin accessibility regulators tend to have higher expression variation. We derived the variance of expression for all transcription factors across micro-arrays after *RMA* normalization and averaging expression values for experiments derived from the cell types. Displayed is a density distribution of the maximal expression variance observed in each subfamily. We partitioned TF subfamilies into two groups depending on whether they had family level CAR ranks of 1 or not. We observe that top ranked subfamilies do have substantially higher variance on average than other subfamilies (linear regression p-value <10^−3^).
(PNG)

S10 Fig Histone-wise motif activities do not substantially associate with TF expression values. H3K4me3 peak data for 51 cell lines were downloaded from ENCODE and histone-wise motif activity was computed and normalized analogously to for DHS data, regressing out the first principal component. We performed the mixed model regression where H3K4me3-based motif accessibility data are regressed on gene expression adding a random effect with the same covariance structure as the expression matrix (denoted ‘histone’). To assess the DHS-independent contribution of H3K4me3 histone activities, we added DHS-based motif accessibility as a covariate (denoted ‘DHS-adjusted histone’). We see that subfamily ranks for both of these strategies do not substantially enrich in low ranks. While ‘histone’ performs mildly better, this is likely due to correlation between the histone activity and DHS activity. In contrast, when DHS-based motif accessibility data was adjusted for H3K4me3-based motif accessibility, we see a still substantial enrichment (see “histone-adjusted DHS” curve). This experiment was performed by regressing DHS motif accessibility on gene expression while adding H3K4me3-based motif accessibilities as a covariate plus a random effect with the same covariance structure as the expression matrix. This shows that of the two activity measures, only DHS activity substantially associates with expression.
(PNG)

S11 Fig *SOX2* expression associates strongly with *POU5F1* motif accessibility. The QQ-plot shows the p-value distribution obtained from the LMM associating the accessibility of the *POU5F1* motif to gene expression values across all genes. We see the strongest association to *SOX2* expression.
(PNG)

S12 Fig precision-recall curves of CAR ranks and PIQ pioneer scores and their combination. Displayed are the precision-recall curves using annotation from Iwafuchi-Doi et al. (2014) as true set. Motif wise PIQ pioneer scores were extracted from Sherwood et al. (2014). For each subfamily, we defined its PIQ pioneer score as the maximal pioneer score for its subfamily members. For 77 subfamilies, data were available from both approaches of which 7 were in the true set. For both CAR ranks and PIQ pioneer scores, precision-recall curves were drawn (CAR rank precision-recall curve starts at 0.43 recall, because many subfamilies share CAR rank of one). Additionally, both scores were combined: For each scoring method, results were ranked (rank ties was replaced by the minimum). For each subfamily, its combined rank is the maximal rank across both methods. A low rank can therefore only be achieved when both methods yielded low ranks. We see that the combined strategy outperforms both base strategies.
(PNG)

S13 Fig Removing first principal component from motif accessibility matrix leads to similar correlation structures between motif accessibility and expression. Displayed are pair-wise correlation matrices with squared entries across cell lines for motif accessibilities (a); motif accessibilities with the first principal component removed (b) and (c) for expression values. Further, the first 25 eigenvalues of these matrices are shown in (d). The motif accessibility matrix has a very dominant first principal component. After removal of the first principal component, the correlation structure of motif accessibility and expression show a similar structure.
(PNG)
