# Supplementary material for: Genome-Wide Association between Transcription Factor Expression and Chromatin Accessibility Reveals Regulators of Chromatin Accessibility
Source: PLoS Comput Biol. 2017 Jan 24;13(1):e1005311. doi: 10.1371/journal.pcbi.1005311 (PMC5261565; doi:10.1371/journal.pcbi.1005311)
Supplement: S1 Appendix — (PDF) [file pcbi.1005311.s001.pdf]

Supplementary Information: Genome-wide association between transcription factor expression and chromatin accessibility reveals regulators of chromatin accessibility.

Supplementary Methods

As mentioned in the main text, we use the following linear mixed model,

$$\boldsymbol{y} = \boldsymbol{x}_i\beta^i + \boldsymbol{\delta}^i + \boldsymbol{\epsilon}^i,$$

where $\boldsymbol{y}$ is a vector of motif accessibility scores across n cell lines, $\boldsymbol{x}_i$ is the expression vector of gene i, β^i is the effect size of gene i:

$$\boldsymbol{\epsilon} \sim N_n(\mathbf{0}, \sigma_r^2 \boldsymbol{I}_n),$$

and

$$\boldsymbol{\delta} \sim N_n(\mathbf{0}, \sigma_e^2 \boldsymbol{C}_e),$$

with

$$\boldsymbol{C}_e = \frac{1}{p}\sum_{i=1}^{p} \boldsymbol{x}_i \boldsymbol{x}_i^T.$$

The likelihood function of is

$$f(\boldsymbol{y}) = \frac{1}{(2\pi)^{n/2}|\sigma_e^2\boldsymbol{C}_e + \sigma_r^2\boldsymbol{I}_n|^{\frac{1}{2}}} \exp\big(-\frac{1}{2}(\boldsymbol{y} - \boldsymbol{x}_i\beta^i)^T(\sigma_e^2\boldsymbol{C}_e + \sigma_r^2\boldsymbol{I}_n)^{-1}(\boldsymbol{y} - \boldsymbol{x}_i\beta^i)\big).$$

We define the spectral decomposition of $\boldsymbol{C}_e$ as:

$$\boldsymbol{C}_e = \boldsymbol{\Gamma}\boldsymbol{\Lambda}\boldsymbol{\Gamma}^T.$$

For any values of σ_e^2 and σ_r^2 we have

$$(\sigma_e^2\boldsymbol{C}_e + \sigma_r^2\boldsymbol{I}_n) = \boldsymbol{\Gamma}(\sigma_e^2\boldsymbol{\Lambda}_e + \sigma_r^2\boldsymbol{I}_n)\boldsymbol{\Gamma}^T,$$

i.e.: the eigenvectors of the mixture matrix are constant w.r.t the mixing parameters. Set

$$\begin{aligned} \boldsymbol{y}' &= \boldsymbol{\Gamma}^T\boldsymbol{y}, \\ \boldsymbol{x}' &= \boldsymbol{\Gamma}^T\boldsymbol{x}. \end{aligned}$$

Since the likelihood is invariant to rotations, we have

$$f(\boldsymbol{y'}) = f(\boldsymbol{y})$$

and

$$f(\boldsymbol{y}') = \frac{1}{(2\pi)^{n/2}|\sigma_e^2\boldsymbol{\Lambda}_e + \sigma_r^2\boldsymbol{I}_n|^{\frac{1}{2}}} \exp\big(-\frac{1}{2}(\boldsymbol{y'} - \boldsymbol{x'}_i\beta^i)^T(\sigma_e^2\boldsymbol{\Lambda} + \sigma_r^2\boldsymbol{I}_n)^{-1}(\boldsymbol{y'} - \boldsymbol{x'}_i\beta^i)\big).$$

Reparametrizing with

$$\gamma = \sigma_r^2/\sigma_e^2,$$

the log-likelihood becomes

$$l(\boldsymbol{y}') = \frac{n}{2}\log(2\pi) - \frac{n}{2}\sum\log(\sigma_e^2(\lambda_i + \gamma)) - \frac{n}{2}\sum\frac{(y_k' - x_{ki}'\beta_i)^2}{(2\sigma_e^2(\lambda_i + \gamma))}$$

Partial derivation shows that the maximum of the log likelihood is reached at

$$\hat{\beta^i} = \sum_k \frac{y_k' x_{ki}'}{(\lambda_k + \hat{\gamma})} / \sum_k \frac{(x_{ki}')^2}{(\lambda_k + \hat{\gamma})},$$

and

$$\hat{\sigma_e^2} = \sum_k \frac{(y_k' - x_{ki}'\beta^i)^2}{(\lambda_k + \hat{\gamma})} / \sum_k \frac{n}{(\lambda_k + \hat{\gamma})}.$$

Reducing the 3 parameter optimization problem to a one parameter optimization over γ. p-values can be obtained by the likelihood ratio test for null hypothesis that $\beta = 0$.
