# Supplementary figures and images for: Genome-Wide Association between Transcription Factor Expression and Chromatin Accessibility Reveals Regulators of Chromatin Accessibility
Source: PLoS Comput Biol. 2017 Jan 24;13(1):e1005311. doi: 10.1371/journal.pcbi.1005311 (PMC5261565; doi:10.1371/journal.pcbi.1005311)

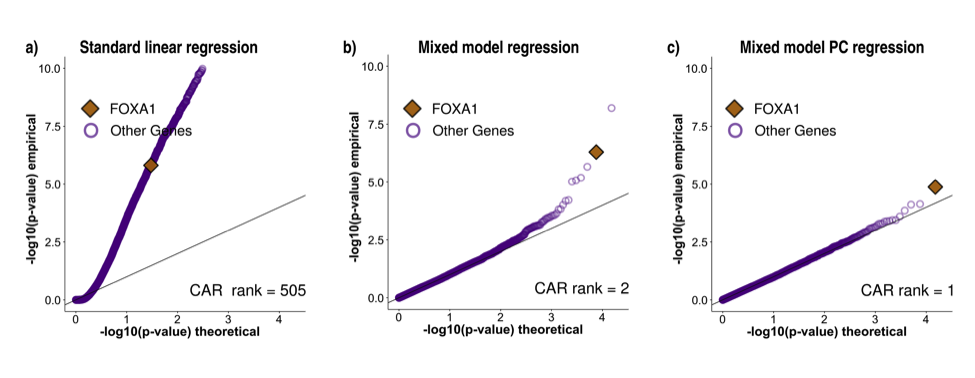

Supplement: S1 Fig — Three different regression models (a-c) were used to compute association p-values between the accessibility of a given TF motif (here FOXA1) and mRNA expression for each of the assayed 15K protein-coding genes. Results are visualized as QQ-plots showing the–log10 transformed p-values. (a) Association p-values obtained using standard linear regression. Due to confounding, p-values are strongly inflated and FOXA1 motif accessibility shows only mild association with FOXA1 expression compared to other genes. (b) The linear mixed model (LMM) successfully corrects for confounding, with most p-values following the null distribution as expected. The association between FOXA1 motif accessibility and FOXA1 expression now ranks second among all genes and first among all TFs, although it does not pass the Bonferroni significance threshold. (c) Additionally controlling for the first principal component of the motif accessibility matrix corrects for a strong batch effect (Methods) and further lowers the CAR rank. Using this approach, FOXA1 motif accessibility showed the strongest association precisely with FOXA1 expression (i.e., the gene-level CAR rank equals one), in line with literature on FOXA1 being a pioneer factor(Cirillo et al. 2002)(Cirillo et al. 2002; Soufi et al. 2015). (PNG) [file pcbi.1005311.s003.png]

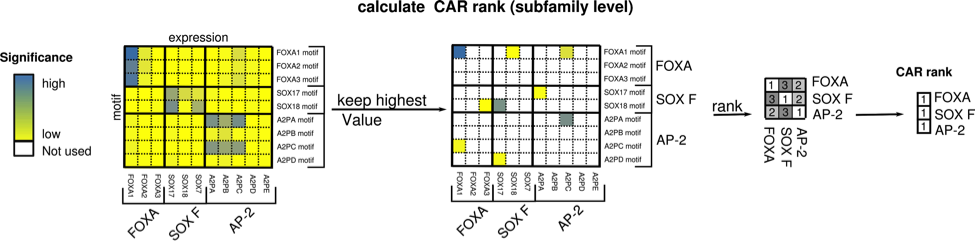

Supplement: S2 Fig — We cluster TFs and motifs according to subfamily definitions given in TFClass. For each bicluster, we define the bicluster score as the most significant p-value between any TF and motif members of the bicluster corrected for bicluster size. We then rank bicluster scores across the TF subfamilies. If the bicluster joining a TF cluster and its corresponding motifs is ranked low, this is an indication of CAR activity. (PNG) [file pcbi.1005311.s004.png]

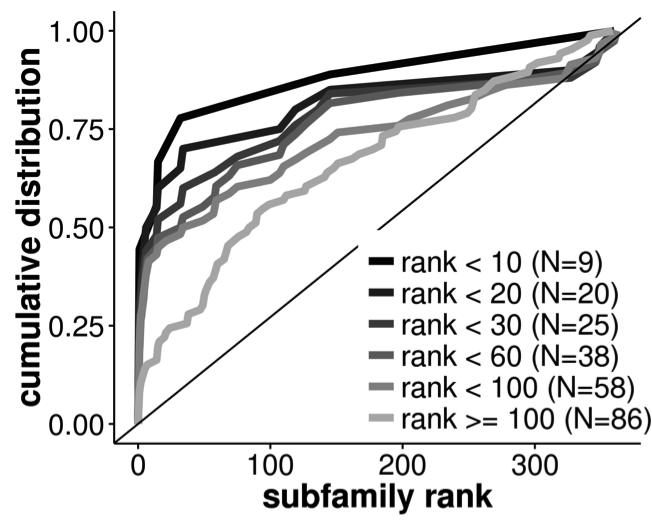

Supplement: S3 Fig — DHS and expression data available for 56 samples (29 with assayed DHS and 27 with imputed DHS) as part of the ROADMAP data collection were used to predict CARs. Shown are CAR enrichment curves for ENCODE results stratified by CAR ranks derived from ROADMAP. Displayed are the following strata: ROADMAP CAR rank <10 (N = 9 observations in total), ROADMAP CAR rank <20 (N = 20 observations in total), ROADMAP CAR rank <30 (N = 25 observations in total), ROADMAP CAR rank <60 (N = 38 observations in total), ROADMAP CAR rank <100 (N = 58 observations in total), ROADMAP CAR rank > = 100 (N = 86 observations in total). We see that subfamilies with low ROADMAP CAR rank also tend to be predicted to be CARs when using the ENCODE data. This enrichment gets weaker for subfamilies with lower ROADMAP CAR ranking. (PNG) [file pcbi.1005311.s005.png]

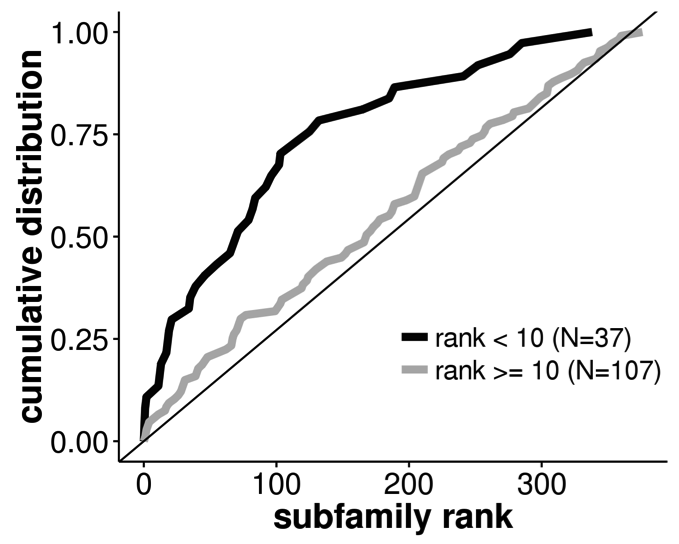

Supplement: S4 Fig — DHS and expression data, available as part of the ROADMAP data collection, were used to predict CARs. Shown are CAR enrichment curves for ROADMAP results stratified by CAR predictions derived from ENCODE. Displayed are the following strata: ENCODE CAR rank <10 (N = 37 observations in total), ENCODE CAR rank > = 10 (N = 107 observations in total). While we see enrichment for low ROADMAP CAR rank in subfamilies predicted to be CARs via the ENCODE data, we see no enrichment in low ROADMAP CAR ranks for other subfamilies. (PNG) [file pcbi.1005311.s006.png]

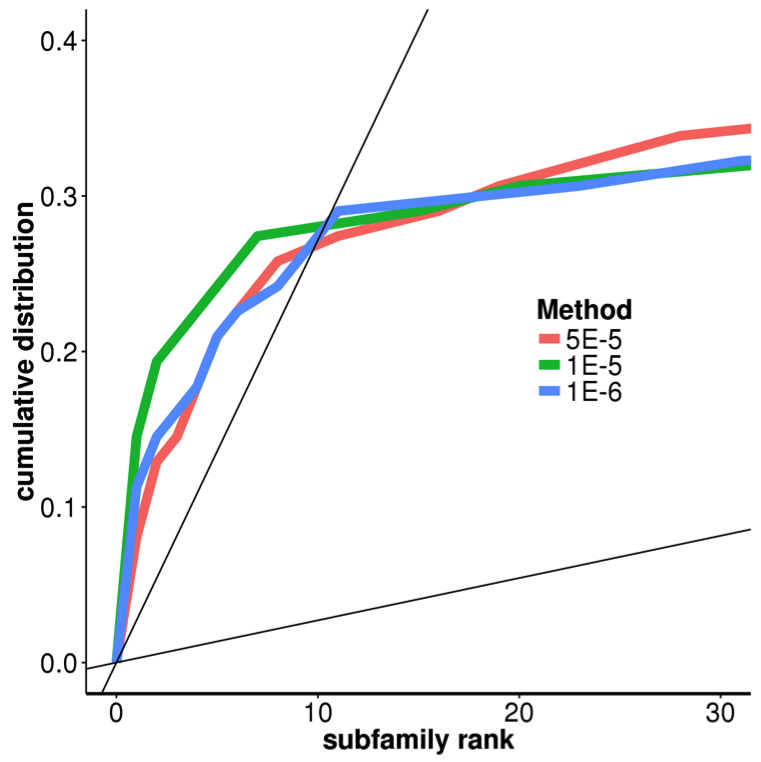

Supplement: S5 Fig — Cumulative distribution of CAR ranks at the subfamily level using the three different motif cutoffs: 10−5 (used throughout the paper) is compared to 10−6 (yielding 9.3 fewer motifs on average [median]) and 5*10−5 (yielding 5.2 more motifs assigned on average). For each setting, we filtered motifs that did not overlap at least 150 DHS regions per cell line on average. Only subfamilies passing this filter in all settings were included (62 subfamilies in total). Power mildly increased at low CAR ranks for more stringent cutoffs at the cost of fewer motifs passing filtering. However, at false discovery rate of 10% power was nearly identical. (PNG) [file pcbi.1005311.s007.png]

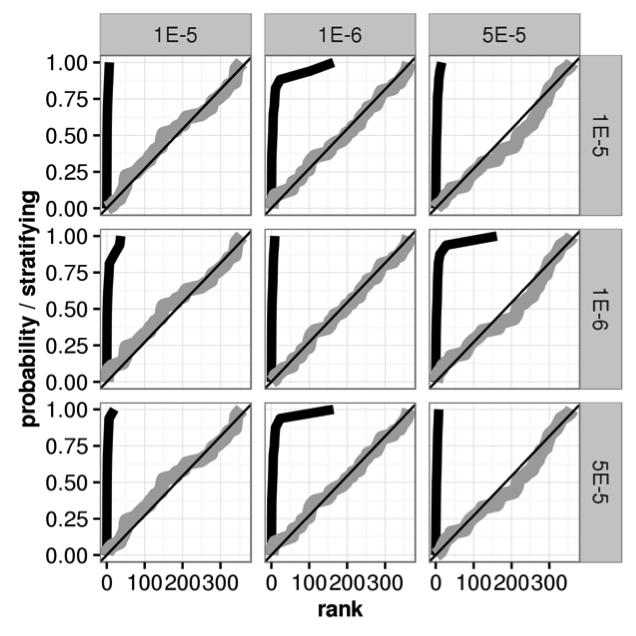

Supplement: S6 Fig — Shown are pairwise comparisons of different motif cutoffs. For each cutoff we derived CAR ranks for all tested subfamilies yielding one CAR rank list per cutoff. Pairwise comparisons of these lists were performed in the following manner: For each pair of rank lists, the first list was used to split the tested subfamilies into a ‘CAR set’ and its complement based on whether a subfamily had CAR rank below 10. For the second results list, two separate CAR enrichment curves were drawn, one curve for the ‘CAR set’ defined via the first list (black) and its complement (grey). Rows denote the cutoff used to derive the ‘CAR set’ and columns denote the cutoff used to draw the enrichment curves. For each setting, we filtered motifs that did not overlap at least 150 DHS regions per cell line on average. Only subfamilies passing this filter in all settings were included (62 subfamilies in total). We see that CARs predicted are stable with respect to varying motif cutoffs. (PNG) [file pcbi.1005311.s008.png]

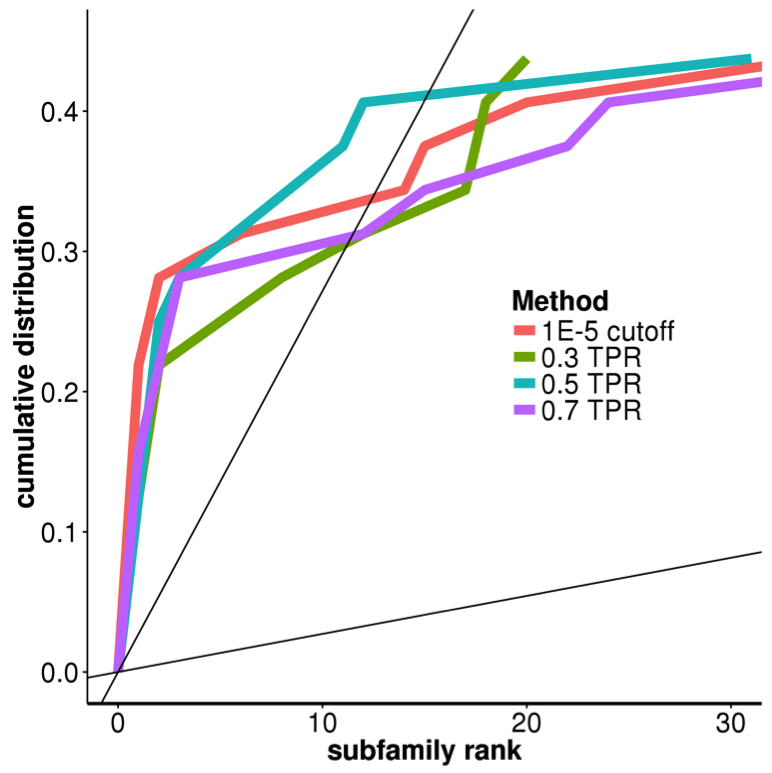

Supplement: S7 Fig — Shown are cumulative distribution of CAR ranks at the subfamily level comparing fixed motif cutoff of 10−5 (used throughout the paper) is compared to variable motif cutoffs guided by ChIP-seq data, where motif cutoffs are adjusted such that called binding sites (i.e. DHS sites containing a motif instance) have a fixed validation rate compared to a gold standard defined by ChiP-seq. Chosen validation rates are 0.3, 0.5 and 0.7. For each setting, we filtered motifs that did not overlap at least 150 DHS regions per cell line on average. Only subfamilies passing this filter in all settings were included (32 subfamilies in total). While we see some variation in power, the variation is not systematic. (PNG) [file pcbi.1005311.s009.png]

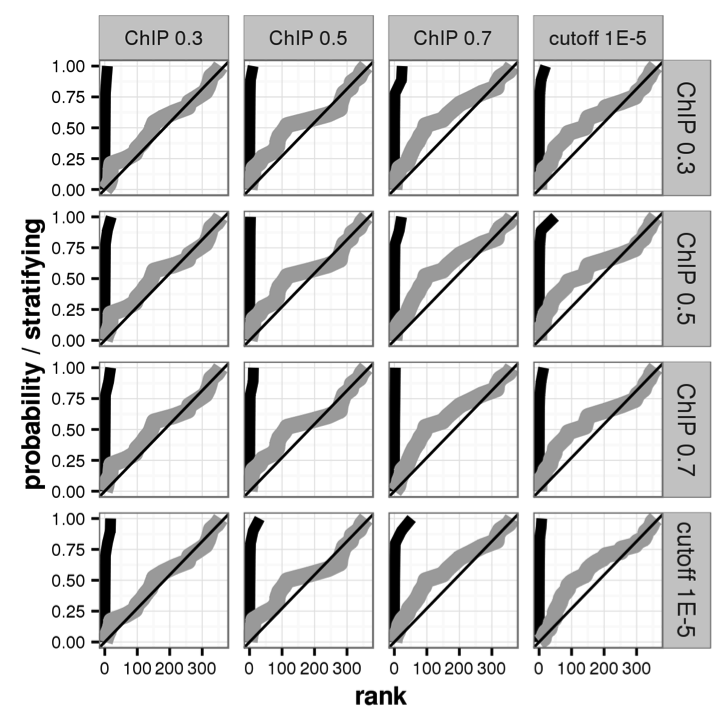

Supplement: S8 Fig — Shown are pairwise comparisons of different motif cutoff methods. For each cutoff method we derived CAR ranks for all tested subfamilies yielding one CAR rank list per method. Pairwise comparisons of these lists were performed in the following manner: For each pair of rank lists, the first list was used to split the tested subfamilies into a ‘CAR set’ and its complement based on whether a subfamily had CAR rank below 10. For the second results list, two separate CAR enrichment curves were drawn, one curve for the ‘CAR set’ defined via the first list (black) and its complement (grey). Rows denote the cutoff method used to derive the ‘CAR set’ and columns denote the cutoff method used to draw the enrichment curves. A fixed motif cutoff of 10−5 (also used throughout the paper) is compared to variable motif cutoffs guided by ChIP-seq data, where motif cutoffs are adjusted such that called binding sites (i.e. DHS sites containing a motif instance) have a fixed validation rate when compared to ChiP-seq. Chosen validation rates are 0.3, 0.5 and 0.7. For each setting, we filtered motifs that did not overlap at least 150 DHS regions per cell line on average. Only subfamilies passing this filter in all settings were included (32 subfamilies in total). We see that CARs predicted are stable with respect to varying motif cutoffs. (PNG) [file pcbi.1005311.s010.png]

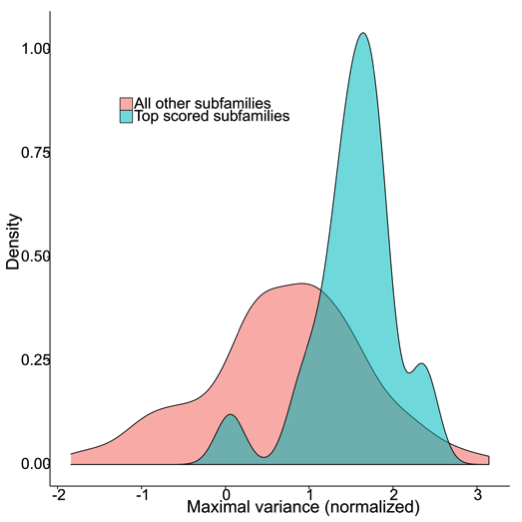

Supplement: S9 Fig — We derived the variance of expression for all transcription factors across micro-arrays after RMA normalization and averaging expression values for experiments derived from the cell types. Displayed is a density distribution of the maximal expression variance observed in each subfamily. We partitioned TF subfamilies into two groups depending on whether they had family level CAR ranks of 1 or not. We observe that top ranked subfamilies do have substantially higher variance on average than other subfamilies (linear regression p-value <10−3). (PNG) [file pcbi.1005311.s011.png]

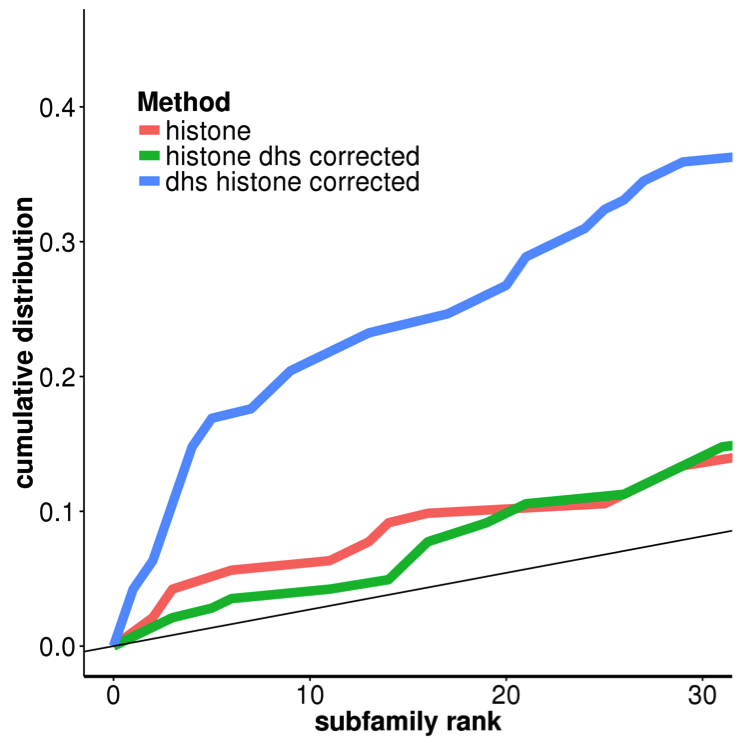

Supplement: S10 Fig — H3K4me3 peak data for 51 cell lines were downloaded from ENCODE and histone-wise motif activity was computed and normalized analogously to for DHS data, regressing out the first principal component. We performed the mixed model regression where H3K4me3-based motif accessibility data are regressed on gene expression adding a random effect with the same covariance structure as the expression matrix (denoted ‘histone’). To assess the DHS-independent contribution of H3K4me3 histone activities, we added DHS-based motif accessibility as a covariate (denoted ‘DHS-adjusted histone’). We see that subfamily ranks for both of these strategies do not substantially enrich in low ranks. While ‘histone’ performs mildly better, this is likely due to correlation between the histone activity and DHS activity. In contrast, when DHS-based motif accessibility data was adjusted for H3K4me3-based motif accessibility, we see a still substantial enrichment (see “histone-adjusted DHS” curve). This experiment was performed by regressing DHS motif accessibility on gene expression while adding H3K4me3-based motif accessibilities as a covariate plus a random effect with the same covariance structure as the expression matrix. This shows that of the two activity measures, only DHS activity substantially associates with expression. (PNG) [file pcbi.1005311.s012.png]

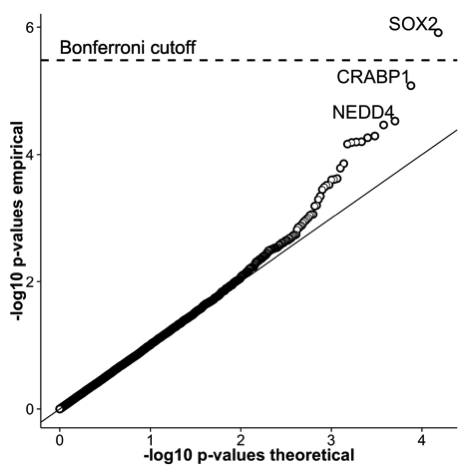

Supplement: S11 Fig — The QQ-plot shows the p-value distribution obtained from the LMM associating the accessibility of the POU5F1 motif to gene expression values across all genes. We see the strongest association to SOX2 expression. (PNG) [file pcbi.1005311.s013.png]

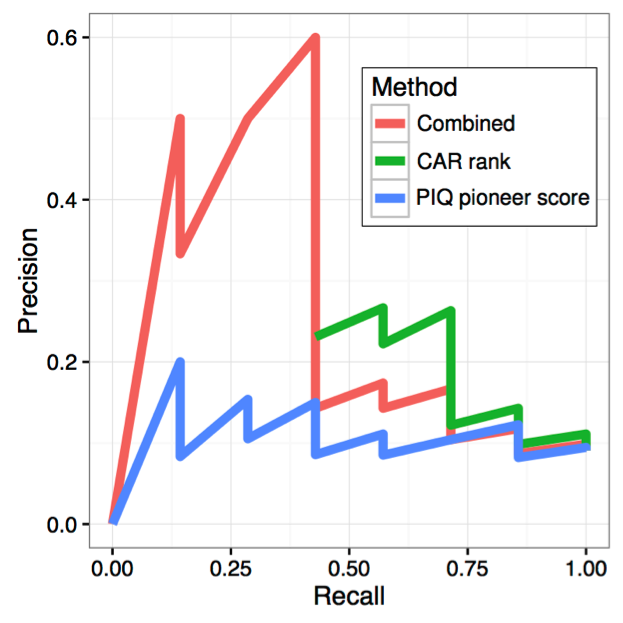

Supplement: S12 Fig — Displayed are the precision-recall curves using annotation from Iwafuchi-Doi et al. (2014) as true set. Motif wise PIQ pioneer scores were extracted from Sherwood et al. (2014). For each subfamily, we defined its PIQ pioneer score as the maximal pioneer score for its subfamily members. For 77 subfamilies, data were available from both approaches of which 7 were in the true set. For both CAR ranks and PIQ pioneer scores, precision-recall curves were drawn (CAR rank precision-recall curve starts at 0.43 recall, because many subfamilies share CAR rank of one). Additionally, both scores were combined: For each scoring method, results were ranked (rank ties was replaced by the minimum). For each subfamily, its combined rank is the maximal rank across both methods. A low rank can therefore only be achieved when both methods yielded low ranks. We see that the combined strategy outperforms both base strategies. (PNG) [file pcbi.1005311.s014.png]

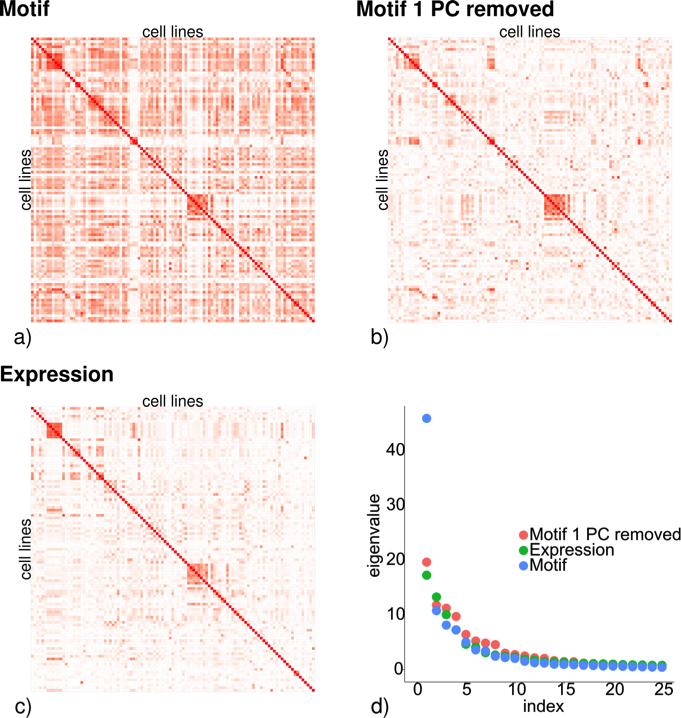

Supplement: S13 Fig — Displayed are pair-wise correlation matrices with squared entries across cell lines for motif accessibilities (a); motif accessibilities with the first principal component removed (b) and (c) for expression values. Further, the first 25 eigenvalues of these matrices are shown in (d). The motif accessibility matrix has a very dominant first principal component. After removal of the first principal component, the correlation structure of motif accessibility and expression show a similar structure. (PNG) [file pcbi.1005311.s015.png]
